# Supplementary material for: A diversified, widespread microbial gene cluster encodes homologs of methyltransferases involved in methanogenesis
Source: bioRxiv. 2023 Sep 26:2023.07.31.551370. Originally published 2023 Jul 31. Preprint. [Version 3] doi: 10.1101/2023.07.31.551370 (PMC10418091; doi:10.1101/2023.07.31.551370)
Supplement: Supplementary file 1 [file media-1.docx]

| Protein name | Annotation | Locus tag | E value | % amino acid identity |
| --- | --- | --- | --- | --- |
| *Cl*Mlp3 | methylcobamide--CoM methyltransferase | CLJU_RS09375 | 4.58e-60 | 32.5 |
| *Cl*UroD7 | methylcobamide--CoM methyltransferase | CLJU_RS10340 | 1.55e-54 | 32.2 |
| *Cl*UroD1 | uroporphyrinogen decarboxylase | CLJU_RS10345 | 2.29e-38 | 30.7 |
| *Cl*UroD8 | methylcobamide--CoM methyltransferase | CLJU_RS10365 | 8.59e-32 | 27.6 |
| *Cl*UroD9 | methylcobamide--CoM methyltransferase | CLJU_RS14945 | 7.10e-29 | 27.0 |
| *Cl*Mlp2 | uroporphyrinogen decarboxylase | CLJU_RS09365 | 9.67e-26 | 27.0 |
| *Cl*Mlp1 | methylcobalamin--coenzyme M methyltransferase | CLJU_RS09360 | 2.57e-10 | 25.8 |

**Table S1**	The results of BLASTp search against the *C. ljungdahlii* DSM 13528 predicted proteome using *M. barkeri* Fusaro MtbA (uniprot ID: O30640) as a query.

**Fig. S1**	Protein SSN of the UroD superfamily (IPR000257). The alignment score cutoff was 40 and the node representation percent cut was 50 % aa identity. Only nodes that are directly or indirectly connected to MGC Mlps are shown for simplicity. Magenta: core type 1 MGC Mlps. Cyan: “auxiliary” type 1 MGC cluster Mlps. Dark Blue: Mlps from non-type 1 MGCs.

**Fig. S2**	**a** Protein SSN of the corrinoid-binding superfamily (IPR006158). The alignment score cutoff was 50 and the node representation percent cut was 50 % aa identity. Only nodes that are directly or indirectly connected to MGC corrinoid proteins are shown for simplicity. Magenta: canonical type 1 MGC corrinoid proteins. Cyan: “auxiliary” type 1 MGC cluster corrinoid proteins that are rarely present in a MGC that already has a canonical corrinoid protein. Dark Blue: corrinoid proteins from non-type 1 MGCs. **b** Protein SSN of the “bacterial-type” Ram/RACE superfamily (IPR027980). The alignment score cutoff was 100 and the node representation percent cut was 50 % aa identity. Only nodes that are directly or indirectly connected to MGC Ram/RACE proteins are shown for simplicity. Magenta: canonical type 1 MGC Ram/RACE proteins. Dark Blue: corrinoid proteins from non-type 1 MGCs.

**Fig. S3**	**Sequence alignments and homology models of the type I MGC Mlps reveal a conserved Zn**^**2+**^ **binding site.** **a** The catalytic role of Zn^2+^ in MtaA and other SCMTs. Coenzyme M (CoM) displaces a conserved carboxylate ligand (Asp or Glu) and is deprotonated, activating the CoM thiolate for nucleophilic attack on the methylated corrinoid protein (MtaC). N and C represent the MtaA N- and C-termini, respectively. **b** Alignments of the Mlps with the positions of the Zn^2+^ ligands indicated with black arrows. The height and color of the bars indicates the extent of amino acid conservation. Tall bars correspond to strongly conserved amino acids, while short bars correspond to low conservation. Alignments were constructed from 147, 268, and 233 sequences encoding Mlps1, 2, and 3, respectively. Green bars represent a completely conserved amino acid while yellow and red bars represent moderately and un-conserved amino acids respectively. Consensus sequences present as simple majority residue representation. **c** A homology model of *Cl*Mlp3 (green) showing the appropriate positioning of the Zn^2+^ (grey sphere) cofactor. *Methanosarcina mazei* MtaA (PDB ID: 4ay7, cyan) was used as a template and is overlayed (cyan). In **b** and **c**, the residue numbering for the *C. ljungdahlii* proteins is shown.

**Fig. S4**	**Absorbance traces of the** ***Cl*****Mlp2 reactions showing steady-state conversion of methylcob(III)alamin to cob(II)alamin with insignificant accumulation of other intermediates.** **a** with sodium sulfide as a methyl acceptor. **b** with sodium selenide as a methyl acceptor. CH_3_-Cbl(III): methylcob(III)alamin. Cbl(II): cob(II)alamin. The reactions are the same as those in Fig 6b.
