# Supplementary material for: A diversified, widespread microbial gene cluster encodes homologs of methyltransferases involved in methanogenesis
Source: bioRxiv. 2023 Jul 31:2023.07.31.551370. Preprint. [Version 1] doi: 10.1101/2023.07.31.551370 (PMC10418091; doi:10.1101/2023.07.31.551370)
Supplement: Supplementary file 1 [file media-1.pdf]

Protein name	Annotation	Locus tag	E value	% amino acid identity
*Cl*Mlp3	methylcobamide--CoM methyltransferase	CLJU_RS09375	4.58e-60	32.5
*Cl*UroD7	methylcobamide--CoM methyltransferase	CLJU_RS10340	1.55e-54	32.2
*Cl*UroD1	uroporphyrinogen decarboxylase	CLJU_RS10345	2.29e-38	30.7
*Cl*UroD8	methylcobamide--CoM methyltransferase	CLJU_RS10365	8.59e-32	27.6
*Cl*UroD9	methylcobamide--CoM methyltransferase	CLJU_RS14945	7.10e-29	27.0
*Cl*Mlp2	uroporphyrinogen decarboxylase	CLJU_RS09365	9.67e-26	27.0
*Cl*Mlp1	methylcobalamin--coenzyme M methyltransferase	CLJU_RS09360	2.57e-10	25.8

Table S1 The results of BLASTp search against the *C. ljungdahlii* DSM 13528 predicted proteome using *M. barkeri* Fusaro MtbA (uniprot ID: O30640) as a query.

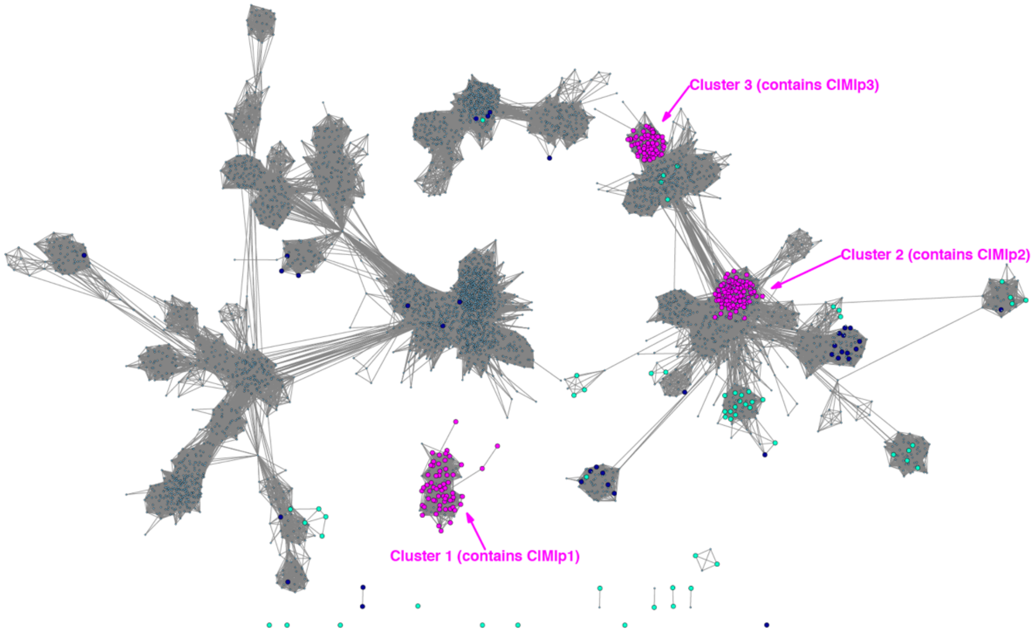

Fig. S1 protein SSN of the UroD superfamily (IPR000257). The alignment score cutoff was 40 and the node representation percent cut was 50 % aa identity. Only nodes that are directly or indirectly connected to MGC Mlps are shown for simplicity. Magenta: core type 1 MGC Mlps. Cyan: “auxiliary” type 1 MGC cluster Mlps. Dark Blue: Mlps from non-type 1 MGCs.

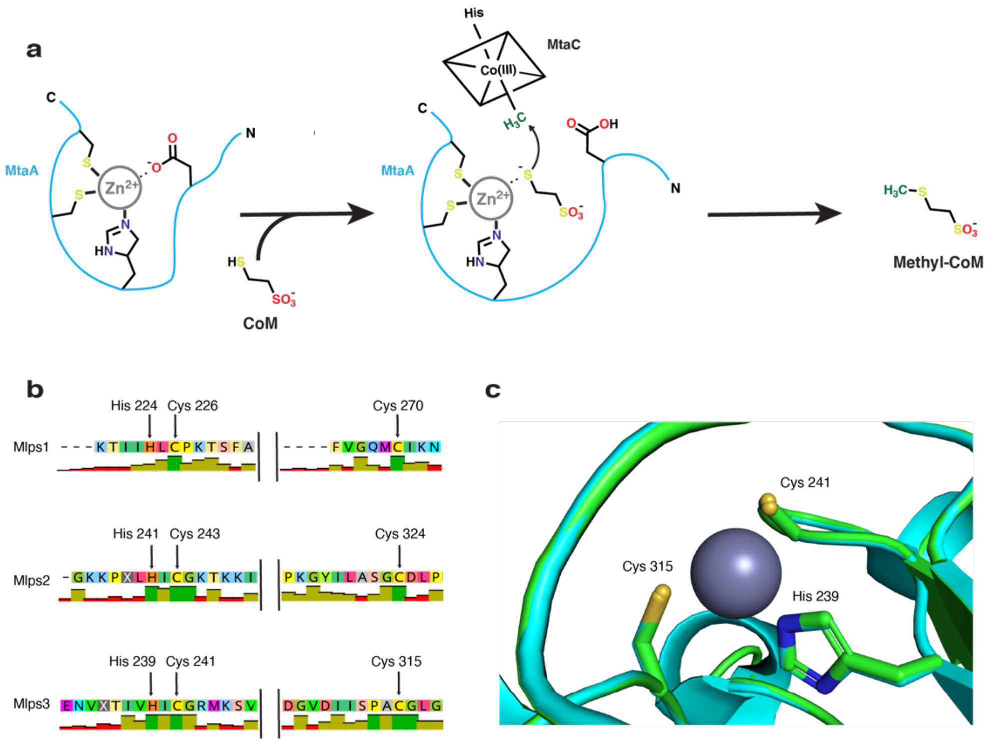

Fig. S2 Sequence alignments and homology models of the type I MGC Mlps reveal a conserved Zn^{2+} binding site. **a** The catalytic role of Zn^{2+} in MtaA and other SCMTs. Coenzyme M (CoM) displaces a conserved carboxylate ligand (Asp or Glu) and is deprotonated, activating the CoM thiolate for nucleophilic attack on the methylated corrinoid protein (MtaC). N and C represent the MtaA N- and C-termini, respectively. **b** Alignments of the Mlps with the positions of the Zn^{2+} ligands indicated with black arrows. The height and color of the bars indicates the extent of amino acid conservation. Tall bars correspond to strongly conserved amino acids, while short bars correspond to low conservation. Alignments were constructed from 147, 268, and 233 sequences encoding Mlps1, 2, and 3, respectively. Green bars represent a completely conserved amino acid while yellow and red bars represent moderately and un-conserved amino acids respectively. Consensus sequences present as simple majority residue representation. **c** A homology model of *Cl*Mlp3 (green) showing the appropriate positioning of the Zn^{2+} (grey sphere) cofactor. *Methanosarcina mazei* MtaA (PDB ID: 4ay7, cyan) was used as a template and is overlayed (cyan). In **b** and **c**, the residue numbering for the *C. ljungdahlii* proteins is shown.

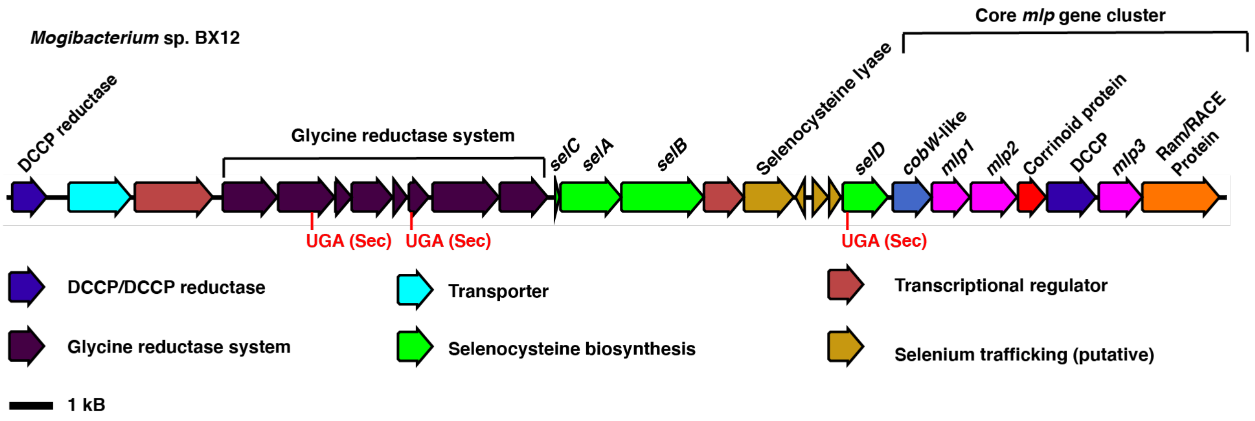

Fig. S3 An example of a type I MGC encoding selenium metabolism genes (*Mogibacterium* sp. BX12). Coloring of the genes encoding the Mlps, corrinoid protein, and Ram/RACE protein are as in Fig. 2 of the main text. Codons for selenocysteine insertion are marked “UGA (Sec)” in red. DCCP: dicubane cluster family protein.

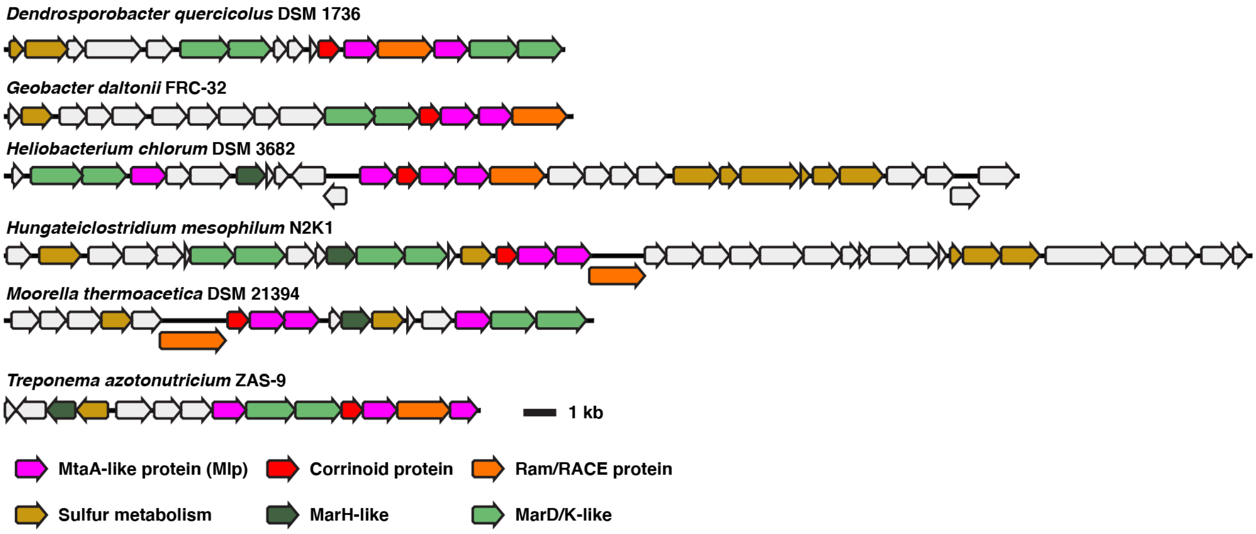

Fig. S4 Selected examples of type I MGCs encoding Mlps 3 from the non-selenium clade, highlighting the co-localization of genes encoding putative methylthioalkane reductase (MarHDK-like) enzymes. The gene clusters selected are from phylogenetically diverse organisms.
